# Sexual Shape Dimorphism of the Mangrove Crab *Ucides cordatus* (Linnaeus, 1763) (Decapoda, Ucididae) Accessed through Geometric Morphometric

**DOI:** 10.1155/2014/206168

**Published:** 2014-10-14

**Authors:** C. E. R. D. Alencar, P. A. Lima-Filho, W. F. Molina, F. A. M. Freire

**Affiliations:** ^1^Grupo de Estudos em Ecologia e Fisiologia de Animais Aquáticos (GEEFAA), Departamento de Biologia, Ecologia e Zoologia, Centro de Biociências, Universidade Federal do Rio Grande do Norte, Campus Universitário Lagoa Nova, Caixa Postal 1524, 59078-970 Natal, RN, Brazil; ^2^Federal Institute of Science and Technology of Rio Grande do Norte, 59500-000 Macau, RN, Brazil; ^3^Centro de Biociências, Departamento de Biologia Celular e Genética, Universidade Federal do Rio Grande do Norte, 59078-970 Natal, RN, Brazil

## Abstract

Sexual dimorphism is often observed in Crustaceans. Considering the great diversity of this subphylum, only few reports are found in the literature and most are mainly based on traditional morphometry. The present study uses geometric morphometrics analysis to identify sexual dimorphism by shape variation in the overexploited semiterrestrial crab *Ucides cordatus*, species with great social and economic importance in South America. Comparative morphology analyses were performed by using the outer face of the propodus of major cheliped, dorsal and anterior region of carapace shape. Significant differences in shape between sexes were detected in these body areas. The causes of dimorphism presented in this species are not clear but, analogous to other possibly associated species, it may be inferred that the causes are with adaptations to body ability of reproductive potential (females), and of reproductive behaviour and agonistics encounters (males). Additional analyses on courtship displays and other reproductive aspects should provide better comprehension of functionality of this morphological differentiation.

## 1. Introduction

Crustaceans comprise a profitable model for morphometric studies, due to the presence of a rigid exoskeleton that allows accurate biometric measurements [1, 2]. The applications of geometric morphometric in crustaceans are numerous, such as using the body shape for taxonomic identifications, fishery stocks, maturity instars, ontogenetic stages, or sexual dimorphism [3–14].

Body shape changes in crabs, either in male or female specimens, can have important ecological consequences and evolutionary trends [15] given that in adults crabs, for instance, physiological processes of growth and reproduction have different targeting energy expenditure for each gender due to the different reproductive activities performed [16]. Hartnoll [16] states that, adaptively, female crabs can show feeding restriction and less number of moults than males during reproductive process; reduced feeding chances are associated with cryptic habits to avoid predation and protect the egg mass, while less number of moults are related to avoiding the loss of the eggs during the incubation period, or, on the other hand, while making the moult, the egg mass would be released with the cast integument and would die due to the absence of parental care.

*Ucides cordatus* (Linnaeus, 1763) [17], a semiterrestrial crab, has great economic importance in Northeast Brazil, where it is considered an overfished species [18]. It inhabits mangroves of the Atlantic coast–from Florida, USA, to Santa Catarina, Brazil [19]–and it is an efficient bioindicator of environmental pollution [20–24]. Curiously, although this species is being exploited during several hundreds of years, many aspects of its biology are scarcely known.

Previous investigations using traditional morphometric methods were performed on populations of* U. cordatus* at South Atlantic coast [25–32] evaluating ontogenetic changes between juveniles and adults of each sex (*see* Hartnoll [33]* for details*) or by simply testing statistically difference on several body measurements between sexes.

However, despite the fact that analyses using geometric morphometric techniques on* U. cordatus* are relatively unknown, they have been used on several crabs genera on sexual dimorphism and evolutionary studies, such as* Liocarcinus *(Linnaeus, 1758) [34], some species of* Uca* (Leach, 1814) [35], and the shrimp* Litopenaeus* (Boone 1931) [4, 10, 14, 15, 36]. Thus, the aim of this study is to investigate the sexual shape dimorphism in three body areas (anterior and dorsal region of carapace and major cheliped) on a* U. cordatus* population from Northeast Brazil based on geometric morphometric analyses.

## 2. Material and Methods

Hundred and twenty specimens of* Ucides cordatus*, 60 of each gender, were used for morphometric analysis. Specimens were obtained in the estuary of Potengi River (05°48′ S, 35°15′ W), state of Rio Grande do Norte, Northeast of Brazil, through active collecting by only one researcher. Immediately after collecting, the animals were placed inside a freezer at −20°C for cryoanesthesia. Identification of specimens was based on Melo [19] and sex classification on the observation of abdominal shape (narrow for males and wide for females) and pleopods number (two pairs in males and four in females). In this study, only specimens with complete appendages without any damage or punctual abnormalities were used. In order to avoid ontogenetic allometry effects [37], only crabs from the same adult cohort (larger sizes from the sexual morphologic maturity size—data not shown) were used. Sizes of sexual morphology maturity were defined by the literature review of previous investigations done in the same geographic region [27].

Digitalized images were obtained by a Sony H10 digital camera (8.1 megapixels) using standardized position and distance. The body areas and structures analysed were the anterior region of cephalothorax (AR), carapace dorsal area (C), and the outer face of major cheliped (MC). Major right cheliped was always used when possible. Image digitalization of chelipeds was facilitated by removing them from the body. This process was carried out cutting them between propodus and carpus [38].

**Figure 1:**
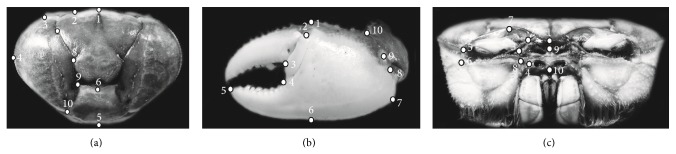
*Ucides cordatus* (Linnaeus, 1763): localization of landmarks in each anatomic area. (a) Dorsal area of carapace—C; (b) outer face of major cheliped—MC; and (c) anterior region of cephalothorax—AR.

The software tpsUtil was used to ordinate the digitalized images in the same file under the TPS format. In addition, the tpsDig2 software [39] was used to record ten landmark in each structure (Figure 1, Table 1). Description of landmarks for AR and C followed anatomical criterion defined by Crane [40] and Williams [41]. Descriptions of landmarks for the MC were based on Rosenberg [3] and Rosenberg [4], with some modifications.* Ucides cordatus* shows a more oval and smooth carapace, without any ornamentation, which makes landmarks establishment difficult, differently from other brachyurans [6, 15]. Thus, the choice of such landmarks was made using intersections of transverse commissures that are strongly marked in this species.

Landmarks coordinates were submitted to a Generalized Procrustes analysis (GPA) [42] in MorphoJ 1.02b [43]. Generalized Procrustes analysis is a procedure that fixes non-shape related variation due to the specimens' position, size, and rotation [44].

In order to avoid static and ontogenetic allometry effects an allometric correction was necessary to compare the body shapes of each gender according to the procedure proposed by Sidlauskas et al. [45]. Thereunto, a pooled within-group allometric regression using centroid size (Size) was performed on Procrustes coordinates (Shape). The statistical significance of the allometric regressions was tested with permutation tests against the null hypothesis of allometry independence [46]. Percentage of predicted allometry in each body area was also calculated as a percentage of total shape variation that the regression model calculated, computed from the Procrustes metric [47, 48]. From this, residuals of the allometric regression were used for statistical analysis and investigation of shape variation [45] for each body part. The definition of static allometry used in this work followed Cock [37], that is, referred to size allometry as a result of the variation of individuals in the same population and age group. In all body area cases, even with static allometry independence (*P* > 0.05), the residuals of the regression were used to obtain further results of shape variation corrected for allometry effect (ontogenetic or static). Subsequent multivariate analysis of variance (MANOVA) was used in order to test shape difference between sexes in each body part separately. A discriminant function analysis (DFA) was used to verify which shape variations could reliably distinguish a gender from the other. Finally, Procrustes distance was inspected to verify which body part showed greater strength in the dissimilarity between genders.

Procedures for allometric correction and multivariate analysis were performed in MorphoJ 1.02b [43]. Moreover, starting from comparative transformation grids (*grids not shown*) obtained from discriminant function, drawing outlines were incorporated to clearly indicate vector variations of mean shape between each gender in each body area. Outline drawing is an alternative form of presentation of the structure under study that makes it easy to interpret shape changes. However, all information provided in this form of representation comes from landmarks. Outline drawing was generated by tpsDig2 [39] and exported to MorphoJ 1.02b [43].

## 3. Results

Landmarks displacements on the body areas (AR, C and MC) revealed significant differences between sexes. Static allometry independence (*P* > 0.05) was detected in AR (*P* = 0.14, predicted = 1.27%) and C (*P* = 0.09, predicted = 1.72%) and allometry dependence was detected in MC (*P* < 0.01, predicted = 5.36%). However, as previously mentioned, for further comparison analysis only residuals of the allometric regression were used. Furthermore, discriminant function analysis ascertains statistical differences in all three body areas revealing major similarity between sexes for AR (Procrustes  distance = 0.0145, *P* < 0.01) and minor for MC (Procrustes  distance = 0.0455, *P* < 0.01) (Table 2). Correct assignments of the cross validation matrix were obtained for MC (98.33% for correct assignment for each sex), C (86.66% for males and 83.33% for females), and AR (71.66% for males and 75.00% for females).

**Figure 2:**
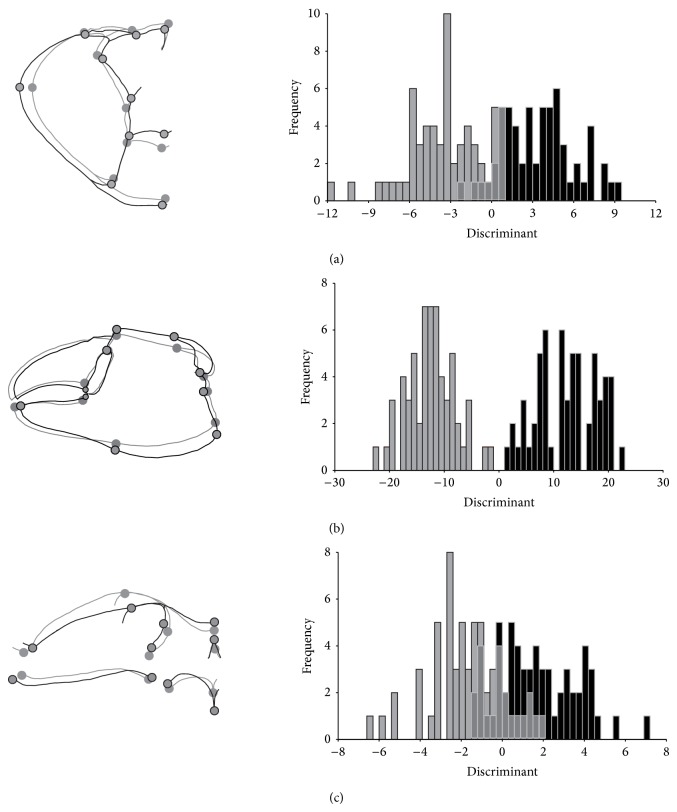
*Ucides cordatus* (Linnaeus, 1763): comparison of body shape from outline drawing; obtained frequencies from Discriminant function analysis. Deviation between male and female correspondent landmarks, in each structure, represents the vector displacement (deformation grid). (a) Dorsal area of carapace—C; (b) outer face of major cheliped—MC; and (c) anterior region of cephalothorax—AR; gray = female and black = male.

Females revealed a less convex profile in C, plus a slight reduction of lateral points and in the anterior region of cephalothorax. On the other hand, points of anterior margin revealed a slight vector displacement on the opposite side. For males, the posterolateral region of C was more rounded than females. Furthermore, points above transversal commissures showed great vector displacement, evidencing a slightly vertical elongation in females with a major functional mesogastric and gonad area. Although, males reveal variation in shape evidencing major functional branchial, gut, and cardiac areas (Figure 2(a)), males presented reduction of the points related to hiatus distance of MC (landmarks 3 and 4) and a sturdy cheliped manus, evidenced by vector displacement of the landmarks in the posterior and inferior margin of the MC. Moreover, the same pattern was observed on the landmarks of the intersection on the base of the dactylus and the articulation between the propodus and carpus. No vector difference was observed on the fixed dactyl shapes (pollex) (Figure 2(b)). Changes in shape for the margin of orbital cavity and its supraorbital margin were meaningful. However, females showed slight vector displacement in the connected landmarks with basis of ocular peduncle (Figure 2(c)).

## 4. Discussion

The three analysed body areas of* U. cordatus* displayed shape variation related to sexual dimorphism. A less convex profile and reduction for anterolateral points found for C in females are probably related to the increase of body ability and reproductive potential [49]. This seems to be a pattern for most brachyurans [6, 11, 15]. During the gonadal development of* U. cordatus *females, this structure increases their volume sometimes reaching four times the initial size. In this condition, upper and lower lobes became evident in the interior of the cephalothorax, occupying a great part of the midgut area. Moreover according to the same authors (see above), the ovarian lobes are connected by a transverse commissure. According to Santana and Silva [50] the transverse commissure displayed a macroscopic morphology which is very similar to gonads forming a structure in the shape of “H” that can be visualized on the dorsal region of* U. cordatus* (indicated by landmarks 6–10; Figure 2(a)). In males, a larger carapace can mean a larger size, or even a more robust basis for the insertion of muscles of pereiopods and chelipeds [15], essential in agonistic encounters in the competition for territory or females [15, 51–53].

Chelipeds of decapods are excellent model for morphologic studies due to their unique structure and the variety of functions [54]. For* Uca pugnax* (Smith, 1870) [55] major chelipeds are related to social behaviour and mating [3]. On the other hand, minor chelipeds are adaptively associated with feeding function. As verified for* Munida rugosa* (Fabricius, 1775) (Galatheidae) [56], chelipeds with higher volume, consequently major finger closing muscle, show marked mechanic advantage [8]. Same conditions are observed for males of* U. cordatus*, considering territorialistic behaviour of this species [49]. In some* U. cordatus* males, some punctual damage or abnormalities beyond several traces were observed confirming the utilization of chelipeds for attack and defence. Previous, traditional morphometric allometric studies with juveniles and adults of* U. cordatus* evidenced a higher ratio of growth for the adult males' chelae than the females', corroborating a potential use in reproductive behavioural display [29].

Based on the aforementioned different uses of chelipeds and knowing that* U. cordatus* males exhibit agonistic behaviour with other males, it may be assumed that, because of the shape variation in chelipeds of* U. cordatus*, the larger hiatus found in chelipeds of females could demonstrate adaptations of its utilization in feeding, while males with smaller hiatus could probably represent major efficiency of the increasing of the prehensile ability that is very important to grab female during the mating and also cause more damage in fights against other males. However, more studies should be realized to confirm this assumption. Morphological differentiations for chelipeds between genders are conspicuously evidenced in size frequency distribution of the discriminant function and the value of the Procrustes distance. This last one pointed it as the major parameter in the differentiation between genders when compared to the other body parts analysed in the present study.

Foraging behaviour, reproduction, and agonistic behaviour constitute a triad of selective pressures that drive evolutionary responses proper for each sex in Brachyura crustaceans [54]. The role of the complex morphologic structures which are evolved in the sexual selection process begins with the shape variation analysis.

Some Brachyurans can show behavioural changes that can be associated with certain body parts. Crabs of the genus* Uca* are a good example for this phenomenon, as males use their hypertrophied cheliped during courtship [40]. Moreover, several evidences indicate the role of such appendage as visual stimulus in conspecific partner choice [57, 58]. Descriptions of mating behavior of* U. cordatus* are still officially unpublished information. However, information from dissertations as well as information obtained during field observations by these authors indicates that* U. cordatus* presents courtship and mating behaviour. It is believed that visual stimulus as coloration and heterochely in males may be decisive for females' acceptation or rejection (*personal communication*).

## 5. Conclusions

Mapping differences of body shape reveal different functions for the same body structures in male and female of the mangrove crab* Ucides cordatus*. These differences are more evident in body parts related with reproductive aspects. In fact, secondary sexual morphologic differences between males and females reveal a sexual dimorphism evolving distinct morphologic aspects of these crabs. In this sense, the present results sustain the hypothesis that morphologic differences found between genders have an important role in sexual selection in this species. Further studies on behavioural aspects could contribute to a better understanding of adaptive functions related with the body structures of this species.

## Figures and Tables

**Table 1:** *Ucides cordatus* (Linaeus, 1763): list of landmarks descriptions used in sexual dimorphism characterization. Dorsal area of carapace—C; Anterior region of cephalotorax—AR and Outer face of major cheliped—MC.

Landmarks	Description
C	
1	Middle point of frontal carapace outer margin
2	Anterolateral margin carapace deviation
3	Intersection of the anterolateral and supraorbital margin
4	Intersection point of anterolateral and posterolateral carapace margin
5	Middle point of posterior carapace margin
6	Middle point of transversal commissure
7	Deviation of anterolateral proportion of transversal commissure
8	Intersection of lateral commissure between hepatic and gastric regions
9	Intersection of lateral commissure between gastric and cardiac regions
10	Posterolateral margin deviation with transversal commissure in intestinal area
AR	
1	Middle point of frontal carapace outer margin
2	Antennula proximal point
3	Antenna proximal point
4	Lateral point of the epistomic margin of carapace
5	Intersection of the lateral margin and supraorbital areas of carapace
6	Distal point of suborbital carapace margin
7	Intersection of supraorbital margin, supraorbital carena, and frontal ramification margin of the transversal commissure
8	Intersection between the suborbital, subhepatic, and pterigostomic regions
9	Superior middle point of the epistomial margin of carapace
10	Inferior middle point of the epistomial margin of carapace
MC	
1	Distal point of the superior manus margin
2	Intersection of the dactyly on superior margin
3	Intersection between the inferior margin of the dactyly and the gap between the pollex and the dactyl
4	Proximal point of the pollex
5	Distal point of the pollex
6	Inferior margin of chelae in rectilinear distance to the distal point of the superior margin
7	Deviation of the inferior margin of the manus
8	Inferior articulation point between the propodus and the carpus
9	Superior articulation point between the propodus and the carpus
10	Proximal deviation of the superior margin of the manus

**Table 2:** *Ucides cordatus* (Linaeus, 1763): statistical results for comparison of shape variation between males and females.

Analysis	Parameters				
MANOVA∗	SS	df	*F*	*P*	Pillai's trace

C	0.0252	16	7.04	<0.01	0.61
AR	0.0049	16	3.61	<0.01	0.43
MC	0.0727	16	25.35	<0.01	0.87

DFA	*T* ^2^	*T* ^2^ *P*	*P* ^**^	*D* ^2^	Prc dist

C	186.67	<0.01	<0.01	2.49	0.0304
AR	86.68	<0.01	<0.01	1.71	0.0145
MC	263.42	<0.01	<0.01	5.04	0.0455

MANOVA, multivariate analysis of variance; DFA, discriminant function analysis; SS, sum square; df, degrees of freedom; *T* ^2^, Hotelling's *t* test; *D* ^2^, Mahalanobis distance; Prc dist, Procrustes distance; dorsal area of carapace, C; outer face of the major cheliped, MC; and anterior region of cephalothorax, AR.

∗Represented only shape results.

∗∗Significance value to permutation tests under Procrustes distance among groups.

## References

[1] Hartnoll R. G. (1978). The determination of relative growth in Crustacea. *Crustaceana*.

[2] Somerton D. A. (1980). A computer technique for estimating the size of sexual maturity in crabs. *Canadian Journal of Fisheries and Aquatic Science*.

[3] Rosenberg M. S. (1997). Evolution of shape differences between the major and minor chelipeds of *Uca pugnax* (Decapoda: Ocypodidae). *Journal of Crustacean Biology*.

[4] Rosenberg M. S. (2002). Fiddler crab claw shape variation: a geometric morphometric analysis across the genus *Uca* (Crustacea: Brachyura: Ocypodidae). *Biological Journal of the Linnean Society*.

[5] Bertin A., David B., Cézilly F., Alibert P. (2002). Quantification of sexual dimorphism in *Asellus aquaticus* (Crustacea: Isopoda) using outline approaches. *Biological Journal of the Linnean Society*.

[6] Rufino M. M., Abelló P., Yule A. B. (2006). Geographic and gender shape differences in the carapace of *Liocarcinus depurator* (Brachyura: Portunidae) using geometric morphometrics and the influence of a digitizing method. *Journal of Zoology*.

[7] Iepure S., Namiotko T., Danielopol D. L. (2007). Evolutionary and taxonomic aspects within the species group *Pseudocandona eremita* (Vejdovský) (Ostracoda, Candonidae). *Hydrobiologia*.

[8] Claverie T., Smith I. P. (2007). Functional significance of an unusual chela dimorphism in a marine decapod: specialization as a weapon?. *Proceedings of the Royal Society B: Biological Sciences*.

[9] Silva I. C., Hawkins S. J., Paula J. (2009). A comparison of population differentiation in two shore crab species with contrasting distribution along the Portuguese coast, using two morphological methodologies. *Marine and Freshwater Research*.

[10] Hopkins M. J., Thurman C. L. (2010). The geographic structure of morphological variation in eight species of fiddler crabs (Ocypodidae: genus *Uca*) from the eastern United States and Mexico. *Biological Journal of the Linnean Society*.

[11] Ledesma F. M., van der Molen S., Barón P. J. (2010). Sex identification of *Carcinus maenas* by analysis of carapace geometrical morphometry. *Journal of Sea Research*.

[12] Silva I. C., Mesquita N., Paula J. (2010). Genetic and morphological differentiation of the mangrove crab *Perisesarma guttatum* (Brachyura: Sesarmidae) along an East African latitudinal gradient. *Biological Journal of the Linnean Society*.

[13] Silva I. C., Alves M. J., Paula J., Hawkins S. J. (2010). Population differentiation of the shore crab *Carcinus maenas* (Brachyura: Portunidae) on the southwest English coast based on genetic and morphometric analyses. *Scientia Marina*.

[14] Accioly I. V., Lima-Filho P. A., Santos T. L., Barbosa A. C. A., Campos L. B. S., Souza J. V., Ara W. C., Molina W. F., Araújo W. C. (2013). Sexual dimorphism in *Litopenaeus vannamei* (Decapoda) identified by geometric morphometrics. *Pan-American Journal of Aquatic Sciences*.

[15] Rufino M., Abelló P., Yule A. B. (2004). Male and female carapace shape differences in *Liocarcinus depurator* (Decapoda, Brachyura): an application of geometric morphometric analysis to crustaceans. *Italian Journal of Zoology*.

[16] Hartnoll R. G. (2006). Reproductive investment in Brachyura. *Hydrobiologia*.

[17] Linnaeus C. (1763). Centuria insectorum. *Amoenitates Academicæ*.

[18] Brasil Proposta do Plano Nacional de Gestão para o Uso Sustentável do caranguejo-uçá, do guaiamum e do siri-azul.

[19] Melo G. A. S. (1996). *Manual de Identificação dos Brachyura (caranguejos e siris) do Litoral Brasileiro*.

[20] Tavares T. M., Beretta M., Costa M. C. (1999). Ratio of DDT/DDE in the All Saints Bay, Brazil and its use in environmental management. *Chemosphere*.

[21] Harris R. R., Santos M. C. F. (2000). Heavy metal contamination and physiological variability in the Brazilian mangrove crabs *Ucides cordatus* and *Callinectes danae* (Crustacea: Decapoda). *Marine Biology*.

[22] do Carmo Fernandes Santos M. (2002). Drinking and osmoregulation in the mangrove crab *Ucides cordatus* following exposure to benzene. *Comparative Biochemistry and Physiology A*.

[23] Nudi A. H., de Luca Rebello Wagener A., Francioni E., de Lemos Scofield A., Sette C. B., Veiga A. (2007). Validation of *Ucides cordatus* as a bioindicator of oil contamination and bioavailability in mangroves by evaluating sediment and crab PAH records. *Environment International*.

[24] Pinheiro M. A. A., Silva P. P. G. E., Duarte L. F. D. A., Almeida A. A., Zanotto F. P. (2012). Accumulation of six metals in the mangrove crab *Ucides cordatus* (Crustacea: Ucididae) and its food source, the red mangrove *Rhizophora mangle* (Angiosperma: Rhizophoraceae). *Ecotoxicology and Environmental Safety*.

[25] Botelho E. R. O., Dias A. F., Ivo C. T. C. (1999). Estudo sobre a biologia do caranguejo-uçá, *Ucides cordatus* cordatus, (Linnaeus, 1763), capturado nos estuários dos Rios Formoso (Rio Formoso) e ilhotas (Tamandaré), no estado de Pernambuco. *Boletim Técnico Científico do CEPENE*.

[26] Ivo C. T. C., Dias A. F., Mota R. I. (1999). Estudo sobre a biologia do caranguejo-uçá, *Ucides cordatus cordatus* (Linnaeus, 1763), capturado no Delta do Rio Parnaíba, Estado do Piauí. *Boletim Técnico-científico do CEPENE*.

[27] Vasconcelos E. M. S., Vasconcelos J. A., Ivo C. T .C. (1999). Estudo sobre a biologia do caranguejo-ucá, *Ucides cordatus* (Linnaeus, 1763), capturado no estuário do rio Curimataú (Canguaretama) no Estado do Rio Grande do Norte. *Boletim Técnico Científico do CEPENE*.

[28] Leite M. M. L., Fonteles-Filho A. A., Silva J. R. F., Cardoso N. S. (2006). Análise do crescimento alométrico no caranguejo-uçá, *Ucides cordatus* (Decapoda: Ocypodidae), no estuário do rio Coreaú, Camocim, Ceará. *Arquivos de Ciências do Mar*.

[29] Pinheiro M. A. A., Hattori G. Y. (2006). Relative growth of the mangrove crab *Ucides cordatus* (Linnaeus, 1763) (Crustacea, Brachyura, Ocypodidae) at Iguape, São Paulo, Brazil. *Brazilian Archives of Biology and Technology*.

[30] Araújo M. S. L. C., Calado T. C. S. (2008). Bioecologia do caranguejo-Uçá *Ucides cordatus* (Linnaeus) no Complexo Estuarino Lagunar Mundáu/Manguaba (CELMM), Alagoas, Brasil. *Revista da Gestão Costeira Integrada*.

[31] Castro A. C. L., Correia M. M. F., Nascimento A. R., Piedade-Júnior R. N., Gama R. L. M., Sousa M. M., Sena A. C. S., Sousa R. C. C. (2008). Aspectos bioecológicos do caranguejo-uçá (*Ucides cordatus cordatus*, L., 1763) (Decapoda, Brachyura) nos manguezais da ilha de São Luís e litoral oriental do Estado do Maranhão, Brasil. *Amazônia: Ciência e Desenvolvimento*.

[32] Goes P., Branco J. O., Pinheiro M. A. A., Barbieri E., Costa D., Fernandes L. L. (2010). Bioecology of the uçá-crab, *Ucides cordatus* (Linnaeus, 1763), in Vitória bay, Espírito Santo State, Brazil. *Brazilian Journal of Oceanography*.

[33] Hartnoll R. G., Wenner A. M. (1985). Growth, sexual maturity and reproductive output. *Factors in Adult Growth*.

[34] Linnaeus C. (1758). *Systema Naturæ*.

[35] Leach W. E. (1814). Crustaceology. *The Edinburgh Encyclopædia*.

[36] Boone L. (1931). A collection of anomuran and macruran crustacea from the bay of panama and the fresh waters of the Canal Zone. *Bulletin of the American Museum of Natural History*.

[37] Cock A. G. (1966). Genetical aspects of metrical growth and form in animals. *Quarterly Review of Biology*.

[38] Silva I. C., Paula J. (2008). Is there a better chela to use for geometric morphometric differentiation in brachyuran crabs? A case study using *Pachygrapsus marmoratus* and *Carcinus maenas*. *Journal of the Marine Biological Association of the United Kingdom*.

[39] Rohlf F. J. tpsDig version 2.10. http://life.bio.sunysb.edu/morph/.

[40] Crane J. (1975). *Fiddler Crabs of the World*.

[41] Williams A. B. (1984). *Shrimps, Lobsters, and Crabs of the Atlantic Coast of the Eastern United States, Maine to Florida*.

[42] Dryden I. L., Mardia K. V. (1998). *Statistical Shape Analysis*.

[43] Klingenberg C. P. (2011). MorphoJ: an integrated software package for geometric morphometrics. *Molecular Ecology Resources*.

[44] Rohlf F. J., Slice D. E. (1990). Extensions of the procrustes method for the optimal superimposition of landmarks. *Systematic Zoology*.

[45] Sidlauskas B. L., Mol J. H., Vari R. P. (2011). Dealing with allometry in linear and geometric morphometrics: a taxonomic case study in the *Leporinus cylindriformis* group (Characiformes: Anostomidae) with description of a new species from Suriname. *Zoological Journal of the Linnean Society*.

[46] Good P. (2000). *Permutation Tests: A Practical Guide to Resampling Methods for Testing Hypotheses*.

[47] Goodall C. R. (1991). Procrustes methods in the statistical analysis of shape. *Journal of the Royal Statistical Society B*.

[48] Klingenberg C. P., Mcintyre G. S. (1998). Geometric morphometrics of developmental instability: analyzing patterns of fluctuating asymmetry with procrustes methods. *Evolution*.

[49] Hartnoll R. G., Bliss D. E. (1982). Growth. *The Biology of Crustacea: Embryology, Morphology and Genetics*.

[50] Santana G. X., Silva J. F. R. (2010). Maturação gonadal em fêmeas do caranguejo-uçá *Ucides cordatus* Linnaeus, 1763 (Decapoda: Brachyura: Ocypodidae) no mangue do Rio Ceará, Caucaia-CE. *Revista da Gestão Costeira Integrada*.

[51] Warner G. F. (1969). The occurrence and distribution of crabs in a Jamaican mangrove swamp. *Journal of Animal Ecology*.

[52] Branco J. O. (1993). Aspectos ecológicos do caranguejo *Ucides cordatus* (Linnaeus, 1763) (Crustacea, Decapoda) do manguezal do Itacorubi, Santa Catarina, Brasil. *Arquivos de Biologia e Tecnologia*.

[53] Nordhaus I., Diele K., Wolff M. (2009). Activity patterns, feeding and burrowing behaviour of the crab *Ucides cordatus* (Ucididae) in a high intertidal mangrove forest in North Brazil. *Journal of Experimental Marine Biology and Ecology*.

[54] Lee S. Y. (1995). Cheliped size and structure: the evolution of a multi-functional decapod organ. *Journal of Experimental Marine Biology and Ecology*.

[55] Ng P. K. L., Guinot D., Davie P. J. F. (2008). Systema Brachyurorum: part I. An annotated checklist of extant brachyuran crabs of the world. *Raffles Bulletin of Zoology*.

[56] Fabricius J. C. Systema entomologiae, sistens insectorvm classes, ordines, genera, species, adiectis synonymis, 
locis, descriptionibvs, observationibvs.

[57] Salmon M., Hyatt G. W., Mccarthy K., Costlow J. D. (1978). Display specificity and reproductive isolation in the fiddler crabs, *Uca panacea* and *U. pugilator*. *Zeitschrift fur Tierpsychologie*.

[58] Detto T., Backwell P. R. Y., Hemmi J. M., Zeil J. (2006). Visually mediated species and neighbour recognition in fiddler crabs (*Uca mjoebergi* and *Uca capricornis*). *Proceedings of the Royal Society B: Biological Sciences*.

